# Molecular Characterization of Plant Volatile Compound Interactions with *Cnaphalocrocis medinalis* Odorant-Binding Proteins

**DOI:** 10.3390/plants13040479

**Published:** 2024-02-07

**Authors:** Qi Qian, Xin Guo, Lingjie Wu, Jiarong Cui, Huiying Gao, Yajun Yang, Hongxing Xu, Zhongxian Lu, Pingyang Zhu

**Affiliations:** 1 College of Life Sciences, Zhejiang Normal University, Jinhua 321004, China; qianq5119@163.com (Q.Q.); gx707343711@163.com (X.G.); 202320500913@zjnu.edu.cn (L.W.); 2019202036@njau.edu.cn (J.C.); ghy2029@163.com (H.G.); luzxmh@163.com (Z.L.); 2 State Key Laboratory for Managing Biotic and Chemical Threats to the Quality and Safety of Agro-Products, Institute of Plant Protection and Microbiology, Zhejiang Academy of Agriculture Sciences, Hangzhou 310021, China; yargiuneyon@163.com

**Keywords:** *Cnaphalocrocis medinalis*, odorant-binding proteins, plant volatiles, binding characteristics, olfactory behavior

## Abstract

Odorant-binding proteins (OBPs) play important roles in the insect olfactory system since they bind external odor molecules to trigger insect olfactory responses. Previous studies have identified some plant-derived volatiles that attract the pervasive insect pest *Cnaphalocrocis medinalis* (Lepidoptera: Pyralidae), such as phenylacetaldehyde, benzyl acetate, 1-heptanol, and hexanal. To characterize the roles of CmedOBPs in the recognition of these four volatiles, we analyzed the binding abilities of selected CmedOBPs to each of the four compounds, as well as the expression patterns of *CmedOBPs* in different developmental stages of *C. medinalis* adult. Antennaes of *C. medinalis* adults were sensitive to the studied plant volatile combinations. Expression levels of multiple *CmedOBPs* were significantly increased in the antennae of 2-day-old adults after exposure to volatiles. *CmedOBP1*, *CmedOBP6*, *CmedPBP1*, *CmedPBP2*, and *CmedGOBP2* were significantly up-regulated in the antennae of volatile-stimulated female and male adults when compared to untreated controls. Fluorescence competition assays confirmed that CmedOBP1 could strongly bind 1-heptanol, hexanal, and phenylacetaldehyde; CmedOBP15 strongly bound benzyl acetate and phenylacetaldehyde; and CmedOBP26 could weakly bind 1-heptanol. This study lays a theoretical foundation for further analysis of the mechanisms by which plant volatiles can attract *C. medinalis*. It also provides a technical basis for the future development of efficient plant volatile attractants of *C. medinalis*.

## 1. Introduction

*Cnaphalocrocis medinalis* (Lepidoptera: Pyralidae) is a major migratory pest that is widely distributed across the world [1,2]. Its primary host is rice, but it also infests important gramineous crops such as barley, wheat, and maize in addition to gramineous weeds such as barnyard grass (*Echinochloa crusgalli*), reed (*Phragmites australis*), and green foxtail (*Setaria viridis*) [3,4]. *Cnaphalocrocis medinalis* has become a major pest in rice in China since the late 1960s [5]. When it is responsible for infestations, it can lead to a reduction in rice production by up to 80% [6]. At present, the prevention and control of *C. medinalis* are achieved primarily through chemical approaches. However, long-term single-pesticide use led to a sharp increase in chemical pesticide resistance among field populations. The export of pesticides produced in China to Southeast Asia has also contributed to a faster resistance gene acquisition both domestically and abroad; annual decreases in chemical pesticide control effects have been observed, leading to *C. medinalis* outbreaks [7,8]. Therefore, it is urgently necessary to develop effective, low-toxicity, and environmentally friendly prevention and control measures. Currently, among the green and non-toxic technical strategies, the most mature application is the use of attractant to lure and kill pests. 

A long period of co-evolution has yielded close relationships between plants and lepidopteran insects. The volatile specialized metabolites released by plants can attract a variety of lepidopteran adults, which then feed on host plant nectar [9]. Compounds that produce significant lepidopteran behavioral activities could be used as efficient attractants in non-toxic insect pest-trapping strategies. This approach would require a detailed understanding of both insect olfactory mechanisms and the behavioral effects of specific plant volatiles on insect pests. 

Odorant-binding proteins (OBPs) are an important class of carrier proteins in insect olfactory sensory lymphatic fluid. The earlier classification is based on different functions: OBPs are divided into general odorant-binding proteins (GOBPs), pheromone-binding proteins (PBPs), and antennal-binding proteins (ABPs) [10,11]. The widespread application of high-throughput techniques such as genomics and bioinformatics has greatly accelerated OBP research; the number of OBP families in various insect orders is constantly increasing [12,13,14,15,16,17]. Vogt et al. (1981) first identified pheromone-binding proteins (PBPs), which bind sex pheromones, in male *Antheraea polyphemus* antennae [16]. The PBPs belong to a specific subclass of OBPs used for sex pheromone detection in Lepidoptera [16]. Subsequently, Breer et al. (1990) discovered another key subclass of OBPs, GOBPs, which can recognize and transport general odor molecules, in the antennae of female *Antheraea pernyi* [10]. However, OBPs are currently categorized into four groups based primarily on the number of conserved cysteines in the OBPs. These are Minus-C OBPs (cysteine residue < 6), Classic OBPs (cysteine residue = 6), Atypical OBPs (cysteine residue ≥ 6), and Plus-C OBPs (cysteine residue > 6 and a highly conserved proline) [17]. In *C. medinalis*, Zeng et al. (2015) identified 30 *OBP* and 26 chemosensory protein (*CSP*) genes [18]. Notably, they found that 12 of these *OBP*s were only expressed in the antennae, and that there were significant sex-specific and age-related differences in *OBP* and *CSP* expression in the antennae [18]. 

Numerous studies confirmed the attractive effects of specific plant volatiles on insects. For example, El-Sayed et al. (2008) identified 19 insect-attractant compounds, including phenylacetaldehyde, methyl salicylate, and benzaldehyde, in a study about *Cirsium arvense* volatiles [19]. Although individual compounds can attract insects [19], mixtures of plant volatile components can enhance such attractive effects; *Aglaomorpha histrio* is trapped with a ternary mixture of eugenol, benzyl acetate, and phenylacetaldehyde as the bait approximately four times more than with phenylacetaldehyde alone [20]. Similar results have been found in *Pseudoplusia includens* and *Dinoderus minutus* [21,22]. Field experiments have confirmed that a combination of four volatiles (phenylacetaldehyde, benzyl acetate, 1-heptanol, and hexanal) significantly attracts *C. medinalis* adults, but the underlying molecular mechanism remained unclear. 

In the present study, we addressed this gap in knowledge by exploring the molecular mechanisms of Classic OBPs of *C. medinalis* to four plant volatile organic compounds (PVOCs). The goals of the study were as follows: (1) to quantify *CmedOBPs* expression patterns in male and female insects across developmental stages and in response to volatile stimulation; and (2) to examine the binding characteristics of four plant volatiles (phenylacetaldehyde, benzyl acetate, 1-heptanol, and hexanal) with CmedOBPs [23]. This study was designed to provide a theoretical basis for the analysis of the mechanisms by which plant volatiles attract *C. medinalis*. Importantly, our findings provide key candidates for influencing *C. medinalis* behavior, ultimately contributing to the development of effective and environmentally friendly control mechanisms for an economically devastating insect pest. 

## 2. Materials and Methods

### 2.1. Insect Rearing

Wheat seedlings were cultured in a growth chamber at 26 ± 1 °C with 60% of relative humidity under a 14/10 h light/dark photoperiod. *C. medinalis* adults were captured from an experimental field in Jinhua City, Zhejiang Province, China (119°38′41″ E; 29°05′33″ N); larvae were reared in an above-growth chamber using wheat as Zhu et al. (2015) described [24]; the pupae were collected in a fresh-keeping box (polypropylene, 205 × 134 × 69 mm) with wet absorbent cotton at the bottom. The top of the box was covered with plastic wrap containing air holes of 10 mm in diameter. Newly emerged adults were collected at 8 PM every night and reared in groups of 12 in disposable plastic cups containing absorbent cotton soaked in 5% honey water for supplementary nutrition. Adult female and male insects were collected at the 1-, 2-, 3-, 4-, and 5-day-old stages in disposable plastic cups. A total of 60 unmated male and female adults of 2 days old were placed in 50 cm × 50 cm × 50 cm cages, respectively. A filter paper containing a mixture of 2 μL of volatiles (mixture with mass ratios of 41.5% phenylacetaldehyde, 36.5% benzyl acetate, 11.2% hexanal, and 10.8% 1-heptanol was dissolved in liquid paraffin) was placed in each cage. After 2 h, insect antennae were collected. This was repeated three times. In the control group, all the conditions were the same as above, except for the use of a filter paper with 2 μL of liquid paraffin.

### 2.2. Electrophysiology

Two-day-old unmated *C. medinalis* individuals (5 males and 5 females) were collected and individually placed under a stereomicroscope. For each insect, the left antenna was carefully removed from the base with a scalpel, then a 1 mm piece was removed from the opposite end. The resulting antenna piece was fixed at both ends of an electrode with conductive glue. Antenna potentiometer (Ockenfels Syntech GMBH, Buchenbach, Germany) was used to measure the potential change, and odor compounds were added after the baseline of the EAG signal was stable. Ten microliters of the volatiles (the same as Section 2.1) and ten microliters of liquid paraffin, as control treatment, were separately dropped onto a 40 × 7 mm piece of filter paper and then inserted separately into a Pasteur pipette as a source of stimulation. The stimulation time was 0.7 s, the stimulation interval was 30 s, and the interval between compounds was 1 min to ensure recovery of antenna sensory function. For each test, the paraffin solution was measured twice before and after the volatile mixture as a control, and the volatile mixture was tested four consecutive times before the control [25]. Relative EAG response values were calculated as follows [25]:

Relative EAG response value = |EAG response| − mean (control measurements)

### 2.3. Total RNA Extraction and cDNA Synthesis

Antennae were collected from 60 pairs of unmated female and male adults with tweezers, then ground in a 1.5 mL nuclease-free centrifuge tube on ice. TRIzol reagent (Thermo Fisher Scientific, Carlsbad, CA, USA) was used to extract the total RNA from the pool of antenna samples according to its instructions. RNA quality and quantity were determined with a NanoDrop 2000 Spectrophotometer (Thermo Fisher Scientific, Waltham, MA, USA). First-strand cDNA was synthesized with a RevertAid First Strand cDNA Synthesis Kit (Thermo Fisher Scientific, Waltham, MA, USA) and stored at −80 °C. 

### 2.4. Real-Time Quantitative PCR (RT-qPCR)

In the results of antennal transcriptome sequencing data of *C. medinalis* [18,26], *CmedOBPs* with full-length genes and specific primers were selected. The accession numbers of the genes were *CmedOBP1* (JN867059), *CmedOBP6* (KP975117), *CmedOBP15* (KP975126), *CmedOBP26* (KX252764), *CmedPBP1* (JN867060), *CmedPBP2*(KC507181), *CmedGOBP1* (JN867057), and *CmedGOBP2* (JN867058). The design and quality evaluation of all primers were completed using Primer-Blast (https://www.ncbi.nlm.nih.gov/tools/primer-blast/ (accessed on 6 June 2021)), and the primer sequence is shown in Table S1. Expression levels of *CmedOBP*s were analyzed in larval samples at multiple instars and in 2-day-old adults with or without exposure to volatiles (the same as Section 2.1). RNA was extracted and cDNA generated as described above (Section 2.3 total RNA extraction and cDNA synthesis). The qPCR was performed on a CFX 96 Real-Time PCR Detection System (Bio-Rad, Hercules, CA, USA). Each 20 μL reaction contained 10 μL of iTaq universal SYBR^®^ Green supermix (Bio-Rad, Hercules, CA, USA), 1 μL of each primer, 1 μL of the target DNA, and 7 μL of RNase-free water. The amplification program consisted of an initial denaturation of 30 s at 95 °C, followed by 35 cycles of 5 s at 95 °C, 30 s at 62 °C, and 5 s at 72 °C. Relative gene expression values were calculated with the 2^−ΔΔCt^ method [27] using β-Actin as the internal control gene. Primers are shown in Table S1. 

### 2.5. In Vitro CmedOBP Expression and Purification

Primers specific for *CmedOBP*s (Table S2) were designed with restriction sites corresponding to the target expression vectors (listed below). Antenna cDNA samples were used as the PCR template for amplification of each gene (Figure S1). Amplicons were purified and recovered using the E.Z.N.A.^®^ Gel Extraction Kit (Omega Bio-Tek, Norcross, GA, USA), then inserted into the cloning vector pMD18-T. Competent *Escherichia coli* DH5α cells (Solarbio Life Science, Beijing, China) were transformed with the resulting constructs. Recombinant plasmids and pET-32a (+) (Solarbio Life Science, Beijing, China) were first digested with the respective restriction enzymes and then ligated with T4 DNA Ligase (TaKaRa, Beijing, China). After the recombinant plasmid was confirmed by sequencing from Beijing Tsingke Biotechnology Company Limited (Beijing, China), competent *E. coli* BL21 (DE3) cells (Solarbio Life Science, Beijing, China) were transformed with the recombinant expression plasmids. Cells carrying the plasmids were selected and cultured in lysogeny broth (LB) to an optical density at 600 nm (OD_600_) of 0.6. Protein expression was induced with the addition of isopropyl β-D-1-thiogalactopyranoside (IPTG) to a final concentration of 0.3 mM. Cells were cultured overnight at 16 °C, then collected via centrifugation (8000 rpm, 4 °C, 10 min). Phenylmethylsulfonyl fluoride was added and broken by an ultrasonic cell disruptor (Ningbo Bcientz, Ningbo, China), which was utilized for a total of 40 min in 3 s intervals with 3 s pauses. After crushing was complete, cells were collected via centrifugation, and the supernatant was added to a HisTrap affinity chromatography column (GenScript, Nanjing, China). Proteins were eluted with a gradient of imidazole buffer (30% and 100%), then detected with sodium dodecyl sulfate (SDS)-polyacrylamide gel electrophoresis (PAGE) and quantified using the Bradford method [28]. Proteins were successfully expressed, except for CmedPBP2.

### 2.6. Fluorescent Competitive Binding Assay

#### 2.6.1. Odorant Preparation and Measurement Parameters

The method for fluorescence competition binding was the same as that described by Zeng et al. (2018) [29]. The fluorescent probe N-phenyl-1-naphthylamine (1-NPN) (Sangon Biotech, Shanghai, China) and phenylacetaldehyde, benzyl acetate, 1-heptanol, and hexanal (Aladdin, Shanghai, China) were diluted to 100 mM stocks in chromatographic-grade methanol and stored at −20 °C. Working solutions of 1 mM 1-NPN and each odorant were made up in methanol. Each CmedOBP solution was diluted in 1 mM Tris-HCl buffer to a final concentration of 2 μM. 

#### 2.6.2. Binding Assays

The RF5301PC fluorescence spectrophotometer (Shimadzu, Japan) was operated at an excitation wavelength of 337 nm and an emission wavelength of 405 nm (with excitation and emission slits of 5 nm and 10 nm, respectively). For each protein solution, 2 mL of diluted protein was added to the quartz cuvette, then 4 μL aliquots of 1-NPN solution were added at 2 min intervals to bring the final concentration of the probe to 2 μM. After the fluorescence intensity was stable, the maximum fluorescence value was recorded. The 1 mM volatile samples dissolved in methanol were then added to a final concentration of 20 μM. After 2 min, when the fluorescence value no longer fluctuated, the maximum fluorescence value was recorded. Each odor sample was tested in three technical replicates. 

#### 2.6.3. Binding Ability Calculations 

The binding ability of each protein to each odorant was determined by plotting the fluorescence intensity against the ligand concentration. Trend lines were linearized with the Scatchard method [30], then used to calculate the half-maximal inhibition concentration (IC_50_) of the ligand. The competition constant was calculated from the IC_50_ value as follows:

Ki = [IC_50_]/(1 + [1-NPN]/K_1-NPN_)

where 1-NPN is the concentration of free 1-NPN, and K_1-NPN_ is the dissociation constant of the CmedOBP/[1-NPN] complex. The dissociation constant (Ki) was inversely correlated with the binding force between the protein and the odorant. At Ki > 50 μmol/L, the protein was considered to have no binding force with the volatile; at 20 μmol/L < Ki < 50 μmol/L, the protein was considered to have a weak binding force with the volatile; and at 0 μmol/L < Ki < 20 μmol/L, the protein was considered to have a strong binding force with the analyzed volatile. 

### 2.7. Statistical Analyses

Statistical analyses were conducted in IBM SPSS Statistics 20 (SPSS Inc., Chicago, IL, USA). Levene’s test for homogeneity of variances was conducted to evaluate the assumption of equality of variances. The EAG data (normally distributed) were analyzed using independent *t*-tests. The qPCR data for *CmedOBP* expression before and after volatile mixture treatment (normally distributed) were analyzed using independent *t*-tests. Comparisons between *CmedOBP* at different days of age were analyzed by one-way ANOVA with Tukey’s honestly significant difference test (Tukey’s HSD). Comparisons between males and females (normally distributed) were made using an independent *t*-test. Differences were considered to be statistically significant at *p* < 0.05.

## 3. Results

### 3.1. C. medinalis EAG Responses to a Combination of Plant Volatiles

**Figure 1:**
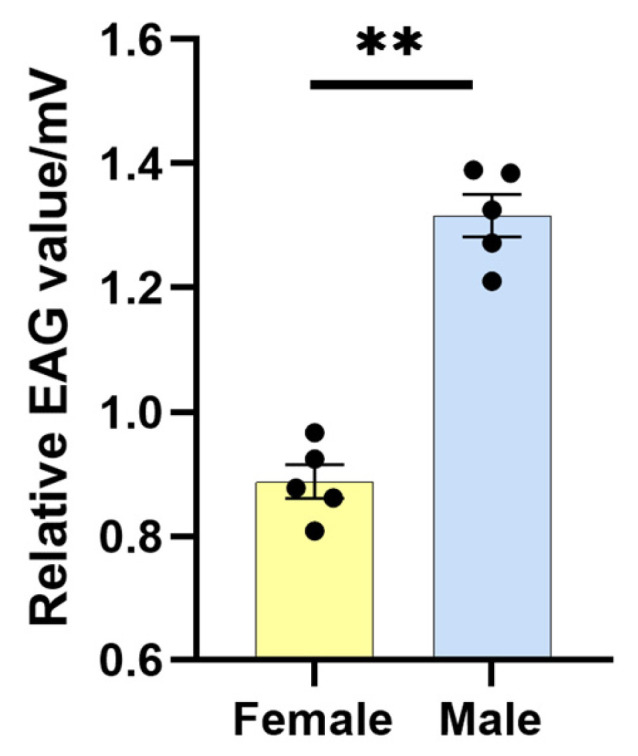
Electroantennogram (EAG) responses of adult male and female *C. medinalis* to a combination of four plant-based volatiles. The mixture was composed of 41.5% phenylacetaldehyde, 36.5% benzyl acetate, 11.2% hexanal, and 10.8% 1-heptanol dissolved in liquid paraffin. Data are shown as the mean ± standard error from five biological replicates (represented by black dots), each containing five insects. Levene’s test showed homogeneity of variance, and then an independent *t*-test was used between female (yellow) and male (blue) insects, ** *p* < 0.01.

To assess the effects of a combination of four plant volatiles, adult male and female *C. medinalis* antennae were exposed to a combination of four plant volatiles. Exposure induced clear electrophysiological responses in both male and female adult antennae. However, the EAG response was significantly higher in male (1.32 ± 0.34 mV) than in female (0.89 ± 0.27 mV) adults (df = 8, F = 0.418, *p* < 0.01) (Figure 1).

### 3.2. Effects of Plant Volatiles on OBP Expression in Adult C. medinalis Antennae

**Figure 2:**
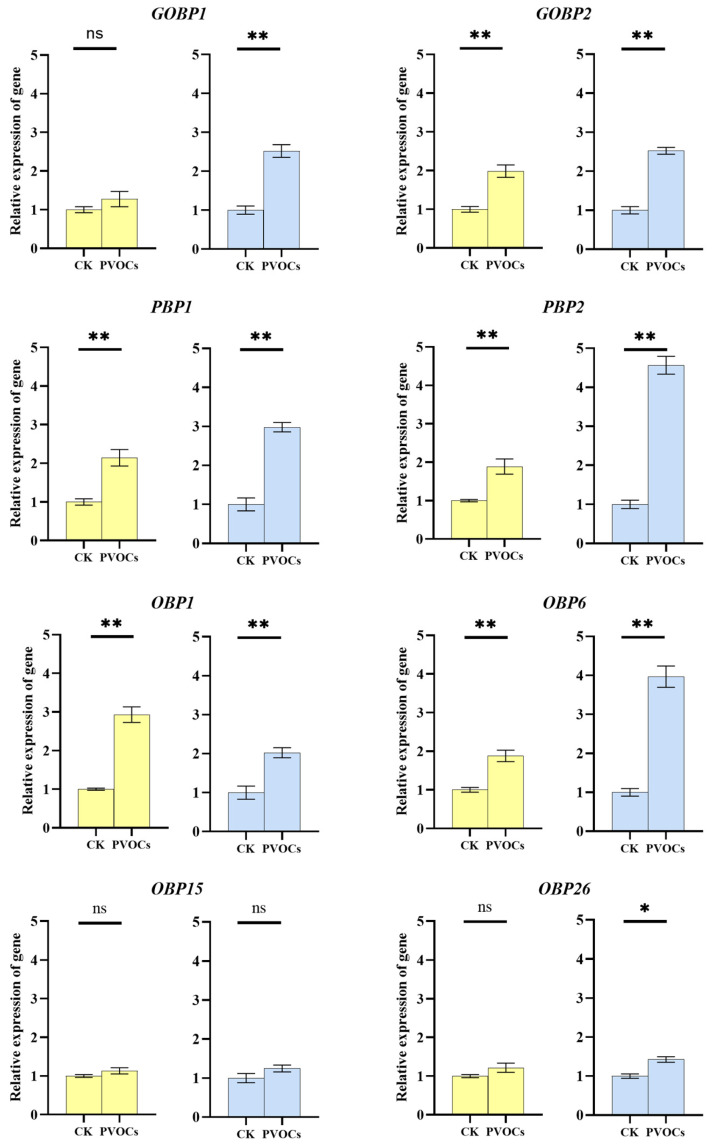
*Odorant-binding protein* (*OBP*) expression in the antennae of adult *Cnaphalocrocis medinalis* after treatment with a combination of four plant-based volatiles. CK, control: insects exposed to the liquid paraffin for 2 h; PVOCs, insects exposed to the volatile mixture (the mixture was the same as Section 3.1) for 2 h. Different column chart colors represent female as yellow, male as blue. * *p* < 0.05, ** *p* < 0.01; ns, no significant difference between CK and PVOCs (independent sample *t*-test).

To establish how volatile exposure affected *OBP*s, expression levels were analyzed in male and female adult insects with and without exposure to the four volatile compounds. Notably, expression levels of some *OBP*s were significantly altered by volatile exposure. Specifically, *CmedOBP1*, *CmedOBP6*, *CmedPBP1*, *CmedPBP2*, and *CmedGOBP2* were significantly up-regulated in both female and male adults (for *CmedOBP1*, female df = 8, F = 2.492, *p* < 0.01 and male df = 8, F = 0.408, *p* < 0.01; for *CmedOBP6*, female df = 8, F = 4.235, *p* < 0.01 and male df = 8, F = 2.116, *p* < 0.01; for *CmedPBP1*, female df = 8, F = 1.506, *p* < 0.01 and male df = 8, F = 0.077, *p* < 0.01; for *CmedPBP2*, female df = 8, F = 4.244, *p* < 0.01 and male df = 8, F = 4.949, *p* < 0.01; for *CmedGOBP2*, female df = 8, F = 1.808, *p* < 0.01; male df = 8, F = 0.152, *p* < 0.01). Furthermore, *CmedOBP26* and *CmedGOBP1* were significantly up-regulated in males (*CmedOBP26*: df = 8, F = 0.009, *p* = 0.02; *CmedGOBP1*: df = 8, F = 1.573, *p* < 0.01) (Figure 2). 

### 3.3. Temporal CmedOBP Expression in Unstimulated C. medinalis Antennae

**Figure 3:**
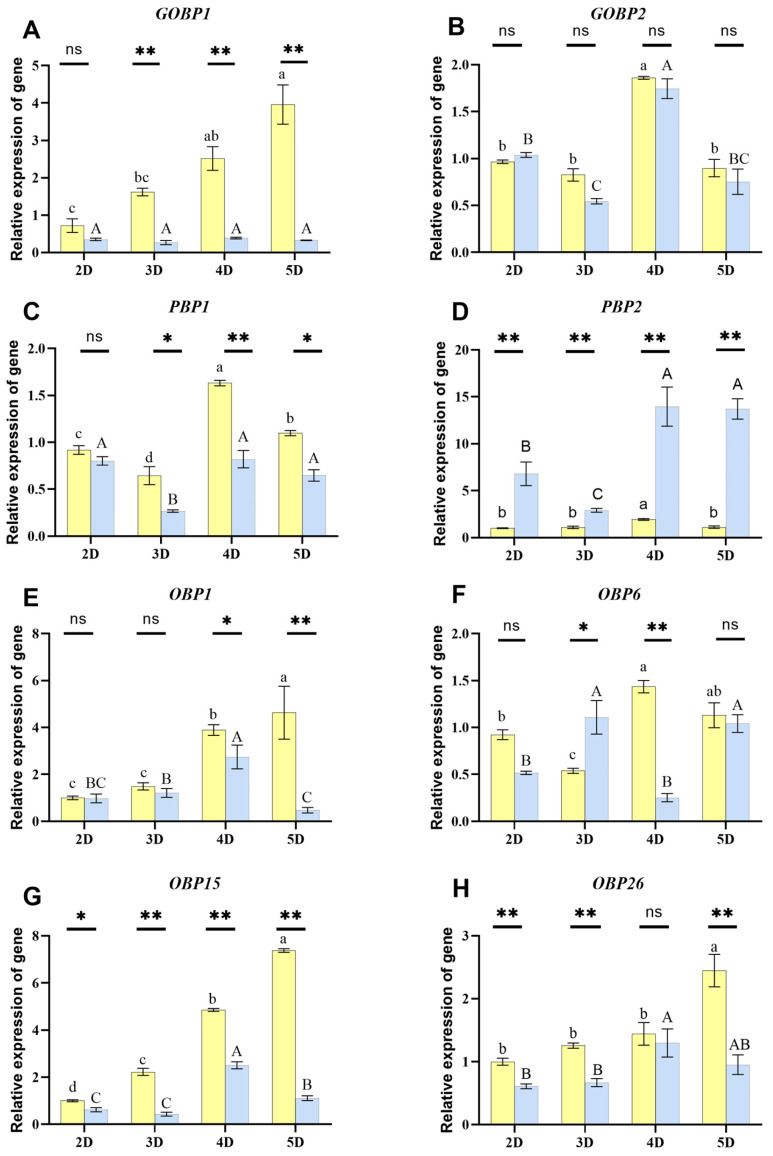
*CmedOBP* expression in the antennae of male and female adults at multiple timepoints after emergence. (**A**–**H**) Expression levels of (**A**) *CmedGOBP1*, (**B**) *CmedGOBP2*, (**C**) *CmedPBP1*, (**D**) *CmedPBP2*, (**E**) *CmedOBP1*, (**F**) *CmedOBP6*, (**G**) *CmedOBP15*, and (**H**) *CmedOBP26*. Uppercase and lowercase letters above each bar indicate statistically significant groups (*p* < 0.05) for male and female insects, respectively, across timepoints (one-way analysis of variance with post-hoc Tukey’s multiple comparison test). Different column chart colors represent female as yellow, male as blue. * *p* < 0.05, ** *p* < 0.01; ns, no significant difference (male compared to female insects at each timepoint for each gene; independent sample *t*-test). Days after emergence (2–5 days) are denoted as 2D–5D, respectively.

We then analyzed the basal *OBP* expression in male and female insects over time. *CmedPBP1*, *CmedPBP2*, *CmedGOBP1*, *CmedGOBP2*, *CmedOBP1*, *CmedOBP6*, *CmedOBP15*, and *CmedOBP26* were expressed in the antennae of both female and male adult *C. medinalis* across timepoints after emergence (2, 3, 4, and 5 d), but with some differences in expression (Figure 3). *CmedGOBP1* was differentially expressed between the antennae of 2-day-old females compared to male adults, but the difference was not statistically significant. However, *CmedGOBP1* was expressed at significantly higher levels in the antennae of female compared to male adults at later ages, particularly at 5 d post-emergence (Figure 3A). The expression of *CmedGOBP2* in the antennae of female and male adults at the same day of age was not significantly different, but the expression levels of *CmedGOBP2* in the antennae of 4-day-old female and male adults were significantly higher than those in other days (*p* < 0.05) (Figure 3B). There were no significant differences in *CmedPBP1* expression between male and female insects at 2 d old, but there were sex-specific expression patterns on the other days of age. *CmedPBP1* peaked in the antennae of 4-day-old females before decreasing but was stably expressed in the antennae of 4- and 5-day-old males (Figure 3C). *CmedPBP2* was expressed at significantly higher levels in the antennae of male compared to female insects and was up-regulated over time, whereas it was expressed at low, stable levels in female antennae. In addition, the relative expression of *CmedPBP2* was the highest in male antennae compared with other genes (Figure 3D). There were no differences in *CmedOBP1* expression between female and male antennae at 2 or 3 d old. However, in the antennae of female individuals, *CmedOBP1* was up-regulated at 4 d old and peaked at 5 d old, whereas levels of this gene peaked at 4 d and decreased again at 5 d in male antennae (Figure 3E). *CmedOBP6* showed a continuous up-regulation over time and significant differences between the antennae of female and male individuals. It showed the highest expression levels in the antennae of 4-day-old female and 3-day-old male individuals (Figure 3F). *CmedOBP15* and *CmedOBP26* were also significantly up-regulated in female compared to male insects at multiple timepoints (Figure 3G,H). In particular, the relative expression of *CmedOBP15* in 2-day-old females continued to increase significantly to 5 d (Figure 3G).

### 3.4. Binding Characteristics of CmedOBPs to Plant Volatiles

To assess the capacity of each CmedOBP to bind the selected plant volatile compounds, fluorescence competitive binding assays were performed with the competitor 1-NPN. Initial assays with the CmedOBPs confirmed that, as expected, fluorescence values first increased along with the 1-NPN concentration, then reached saturation (Figure S2). The concentration of bound 1-NPN was evaluated based on the fluorescence intensity value, assuming that the protein exhibited 100% activity at saturation, with a stoichiometric ratio of 1:1 (protein: ligand). 

**Figure 4:**
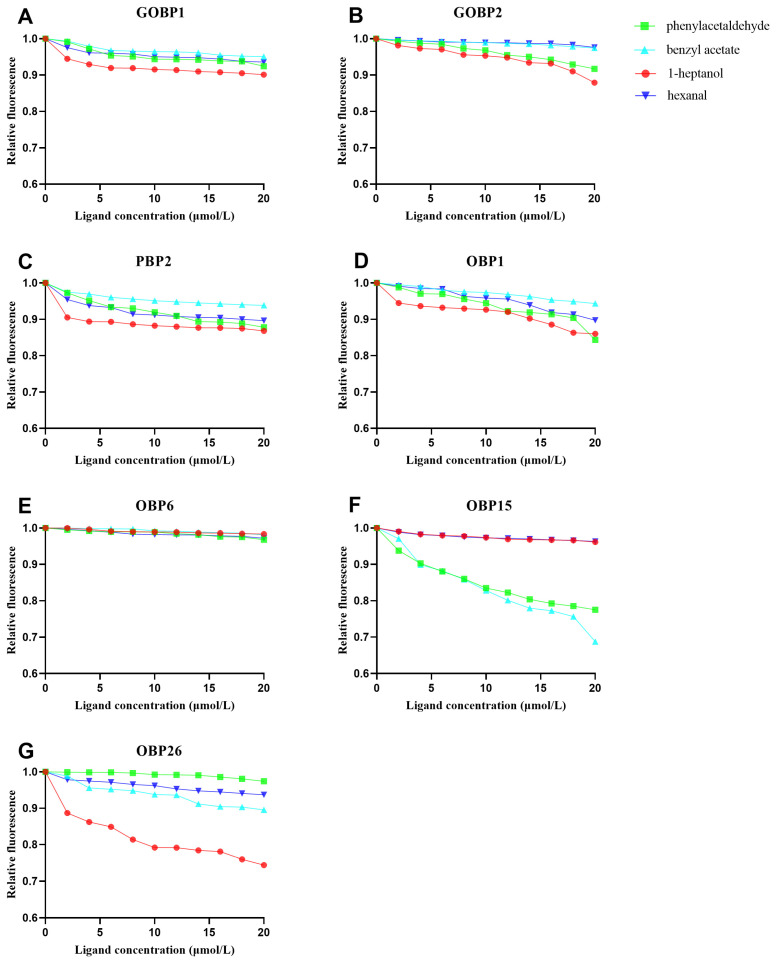
Competitive binding between plant-based volatiles and N-phenyl-1-naphthylamine (1-NPN)–*Cnaphalocrocis medinalis* odorant-binding proteins (CmedOBPs). (**A**–**G**) Competition binding curves of (**A**) CmedGOBP1, (**B**) CmedGOBP2, (**C**) CmedPBP2, (**D**) CmedOBP1, (**E**) CmedOBP6, (**F**) CmedOBP15, and (**G**) CmedOBP26 with phenylacetaldehyde, benzyl acetate, 1-heptanol, and hexanal.

Further fluorescence binding assays were used to calculate the IC_50_ and Ki values of the CmedOBPs for four plant volatiles (Table 1). The Ki values of CmedPBP2, CmedGOBP1, CmedGOBP2, and CmedOBP6 were >50 μM for all four volatiles, indicating a total lack of binding ability. The Ki values of CmedOBP15 with benzyl acetate and phenylacetaldehyde were <20 μM, corresponding to a strong binding ability (Figure 4F). CmedOBP26 had a Ki value between 20 μM and 50 µM for 1-heptanol, indicating a weak binding force (Figure 4G). The Ki values of CmedOBP1 for 1-heptanol, phenylacetaldehyde, and hexanal were all <20 μM, demonstrating strong binding capacity (Figure 4D). 

## 4. Discussion

### 4.1. EAG and CmedOBP Expression Responses to Plant Volatile Exposure

Although plants can release a huge number of volatile compounds, only a few of these cause insect-attractant effects [31]. Despite the significant responses to plant volatiles that insects exhibit, the underlying molecular mechanisms are largely unknown [32]. We used EAG here to directly determine whether a combination of plant volatiles could cause electrophysiological responses in *C. medinalis* antennae. Indeed, the adult *C. medinalis* antennae were sensitive to the examined plant volatile combinations, with both females and males having electrophysiological responses. 

EAG only represent the overall activity of all of the sensilla on the antenna. To further understand the putative molecular functions of specific *CmedOBPs*, gene expression levels were analyzed after exposure to volatile compounds. Some *CmedOBPs* were significantly up-regulated after volatile stimulation of the antennae, and there were also significant differences in antennae expression between male and female adults. *CmedOBP15* showed consistent expression levels between control antennae and those exposed to volatiles, and it may not be the main functional protein in the antennae to transport the volatile mixture. *CmedOBP1*, *CmedOBP6*, *CmedPBP1*, *CmedPBP2*, and *CmedGOBP2* were significantly up-regulated in both male and female antennae following volatile stimulation, suggesting that each of these genes played a role in transporting at least one of the four tested odor molecules. The up-regulation of *CmedOBP26* and *CmedGOBP1* in the antennae of only male insects after volatile stimulation suggested that the corresponding proteins may have had male-specific functions in the olfactory system. Host plant volatiles have been shown to synergistically enhance the stimulatory effects of sex pheromones [33,34]; thus, the significant up-regulation of both *CmedOBP26* and *CmedGOBP1* in the antennae of males after stimulation led us to hypothesize that these genes functioned in male recognition of sex pheromones. 

### 4.2. Gene Expression Analysis of CmedOBPs 

Insect olfactory systems allow for the recognition of trace volatiles from hosts or mates and the avoidance of toxic compounds or natural enemies [35]. OBPs are critical proteins that bind odor molecules during olfactory perception and transport odor molecules across the lymphatic environment [36]. OBPs transport specifically bound odor molecules to olfactory receptors to form olfactory signals, so that insects can accurately and precisely discriminate between potential mates and identify host plants [37,38]. However, insect OBPs are also responsible for specific chemical signal recognition in the olfactory sensory system [39]. 

The functions of insect receptors are influenced by numerous factors, including age, developmental stage, and nutritional status [40]. Clarifying expression levels of OBPs in the antennae of adult insects of different ages and genders, taking into account the physiological characteristics of these insects, can provide information about the mechanisms by which OBPs produce specific behavioral responses. In the present study, we investigated the possible functions of OBPs in the antennae of adult male and female *C. medinalis* individuals. Clear temporal and sex-specific expression patterns were identified. For example, *CmedGOBP1*, *CmedOBP1*, *CmedOBP15*, and *CmedOBP26* were highly expressed in the antennae of adult female *C. medinalis* at multiple ages. We therefore hypothesize that the encoded proteins play a crucial role in facilitating the transport of lipid-soluble substances through the antennal lymph, which is essential for female-specific behaviors, such as host plant identification and oviposition site selection. However, the specific role of these proteins in these behaviors remains to be determined and requires further investigation. Female pheromones are important components of sexual chemical communication in lepidopteran insects [41]; *CmedPBP2* was more highly expressed in the antennae of male than female individuals at all ages, suggesting that *CmedPBP2* may have been involved in male recognition of female sex pheromones. There was no significant difference in the antennae of *CmedGOBP2*, *CmedPBP1*, and *CmedOBP6* between females and males. We hypothesized that the encoded proteins were involved in basic, conserved olfactory functions common to both female and male insects, such as food finding and recognition of common volatiles. The higher expression of many *CmedOBP*s in females than in males is consistent with prior findings that female insects are more sensitive to the host environment [42,43]. 

### 4.3. CmedOBP–Plant Volatile Binding Characteristics

OBPs can transport chemicals to odorant receptors (ORs), which comprise the first step in insect olfactory molecule recognition [44]. As demonstrated here and in prior publications, OBP functions can be delineated through a combination of fluorescence competition binding, EAG assays, and RNA interference (RNAi) [45]. For example, *Bradysia odoriphaga* shows strong EAG responses to the host plant volatiles methyl allyl disulfide and diallyl disulfide. Fluorescence competitive binding has shown that BodoOBP10 binds strongly to these two host plant volatiles, and RNAi has further indicated that BodoOBP10 is involved in host plant recognition and localization [46,47]. In *Sitobion avenae*, the OBP SaveOBP9 shows a strong binding force (Ki < 10 μM) with four wheat compounds: tetradecane, octanal, decanal, and hexadecane [48]. *S. avenae* also shows marked behavioral responses to these four compounds. SaveOBP9 may therefore function in wheat volatile identification and transport, and it is a valuable candidate for comprehensive *S. avenae* management [48]. Similar management strategies could be applied for the control of *C. medinalis* using plant volatiles with insect-attractant properties and strong binding forces with CmedOBPs. 

In lepidopteran species, OBPs are considered to mainly sense odor molecules, including those emitted by host plants, foods, and other sources of volatile substances [49,50,51]. The *C. medinalis*-attracting compounds hexanal and 1-heptanol are commonly found in rice leaves [52,53], whereas phenylacetaldehyde and benzyl acetate are most often found in floral organs; the latter compounds are broad-spectrum insect attractants that guide long-distance localization of moths [54,55]. We found here that CmedOBP1 had strong binding abilities with 1-heptanol, phenylacetaldehyde, and hexanal but not with benzyl acetate. Fluorescence binding assays indicated that CmedOBP1 may have important roles in *C. medinalis* for the specific perception of 1-heptanol, phenylacetaldehyde, and hexanal (Figure 4D). CmedOBP15 could strongly bind with benzyl acetate and phenylacetaldehyde (Figure 4F), indicating that this protein may be responsible for identifying and transporting these two types of plant volatiles. Although binding of these plant volatiles by CmedOBPs was clearly shown, subsequent *C. medinalis* behavioral effects were not determined and will require additional study. Furthermore, CmedGOBP1, CmedGOB2, CmedPBP2, and CmedOBP6 did not bind to any of the four examined volatiles; the specific ligands of these OBPs should thus be further explored.

## 5. Conclusions

Here, we found that a combination of plant volatiles significantly up-regulated *CmedOBP1*, *CmedOBP6*, *CmedPBP1*, *CmedPBP2*, and *CmedGOBP2* in the antennae of *C. medinalis*. The up-regulation effect of exposure to volatiles on *CmedGOBP1* and *CmedOBP26* was observed only in males. Furthermore, our results preliminarily demonstrated sex-specific *CmedOBP* expression. It was indicated that CmedOBP1, CmedOBP15, and CmedOBP26 specifically participate in the identification, binding, and transportation of one or several plant volatiles such as benzyl acetate, phenylacetaldehyde, 1-heptanol, and hexanal in *C. medinalis* antennae. Overall, these results provide a theoretical basis for further exploration of the mechanisms by which plant volatiles attract female and male *C. medinalis* adults and of the functions performed by CmedOBPs in olfactory perception. Most importantly, our findings suggest key candidate compounds for the future development of precise, effective, environmentally responsible *C. medinalis* control.

## Figures and Tables

**Table 1:** Binding forces between *Cnaphalocrocis medinalis* odorant-binding proteins (CmedOBPs) and four plant-based volatiles.

CmedOBP	1-Heptanol		Benzyl Acetate		Phenylacetaldehyde		Hexanal	
	IC_50_ μmol/L	Ki μmol/L	IC_50_ μmol/L	Ki μmol/L	IC_50_ μmol/L	Ki μmol/L	IC_50_ μmol/L	Ki μmol/L
CmedGOBP1	219.2	>50	223.1	>50	161.8	>50	249	>50
CmedGOBP2	99.7	>50	454.3	>50	123.0	>50	622.1	>50
CmedPBP2	223.9	>50	235.3	>50	85.8	>50	154.7	>50
CmedOBP1	68.1	11.99	178.6	31.45	77.5	13.65	99.2	17.46
CmedOBP6	623.5	>50	558	>50	356.8	>50	382.6	>50
CmedOBP15	289.9	>50	34.4	13.61	44.3	17.53	441.2	>50
CmedOBP26	52.3	31.89	96.1	>50	458.1	>50	199.9	>50

## Data Availability

Data is contained within the article and supplementary materials.

## Supplementary Materials

The following supporting information can be downloaded at: https://www.mdpi.com/article/10.3390/plants13040479/s1, Figure S1: Identification of amplification bands of *Cnaphalocrocis medinalis* odorant binding proteins genes by agarose gel electrophoresis; Figure S2: The binding curves and the Scatchard equations of N-phenyl-1-naphthylamine (1-NPN) and *Cnaphalocrocis medinalis* odorant binding proteins (CmedOBPs); Table S1: RT-qPCR primer sequences of *Cnaphalocrocis medinalis* odorant binding proteins genes; Table S2: RT-PCR primers for *Cnaphalocrocis medinalis* odorant binding protein genes.

## References

[1] Khan Z., Barrion A., Litsinger J., Castilla N., Joshi R. (1988). A bibliography of rice leaffolders (Lepidoptera: Pyralidae). Insect Sci. Its Appl..

[2] Xu J., Li C.M., Yang Y.J., Qi J.H., Zheng X.S., Hu R.L., Lu Z.X., Liu Q. (2012). Growth and reproduction of artificially fed *Cnaphalocrocis medinalis*. Rice Sci..

[3] Chintalapati P., Gururaj K., Vallabuni S., Yenumulag P. (2015). Physiological age status of female adults and off-season survival of rice leaffolder *Cnaphalocrocis medinalis* in India. Rice Sci..

[4] Khan Z.R., Abenes M.L.P., Fernandez N.J. (1996). Suitability of graminaceous weed species as host plants for rice leaffolders, *Cnaphalocrocis medinalis* and *Marasmia patnalis*. Crop Prot..

[5] Yang Y.J., Xu H.X., Zheng X.S., Tian J.C., Lu Y.H., Lu Z.X. (2015). Progresses in management technology of rice leaffolders in China. J. Plant Prot..

[6] Shah S.M.A., Hidayat-ur-Rahman, Rehman A., Abassi F.M., Khalil I.H., Ali A. (2008). Characterization of wild rice species in response to leaffolder *Cnaphalocrocis medinalis*. Sarhad J. Agric..

[7] Xu J., Liu Q., Li C.M., Han G.J. (2019). Field effect of *Cnaphalocrocis medinalis* granulovirus (CnmeGV) on the pest of rice leaffolder. J. Integr. Agric..

[8] Sun Y., Liu S., Ling Y., Wang L., Ni H., Guo D., Dong B., Huang Q., Long L., Zhang S. (2023). Insecticide resistance monitoring of *Cnaphalocrocis medinalis* (Lepidoptera: Pyralidae) and its mechanism to chlorantraniliprole. Pest Manag. Sci..

[9] Kevan P.G., Baker H.G. (1983). Insects as flower visitors and pollinators. Annu. Rev. Entomol..

[10] Breer H., Krieger J., Raming K. (1990). A novel class of binding proteins in the antennae of the silk moth *Antheraea pernyi*. Insect Biochem..

[11] Zhou J.J., Robertson G., He X., Dufour S., Hooper A.M., Pickett J.A., Keep N.H., Field L.M. (2009). Characterisation of *Bombyx mori* odorant-binding proteins reveals that a general odorant-binding protein discriminates between sex pheromone components. J. Mol. Biol..

[12] Zhou J.J. (2010). Odorant-binding proteins in insects. Vitam. Horm..

[13] Leal W.S. (2013). Odorant reception in insects: Roles of receptors, binding proteins, and degrading enzymes. Annu. Rev. Entomol..

[14] Pelosi P., Iovinella I., Zhu J., Wang G., Dani F.R. (2018). Beyond chemoreception: Diverse tasks of soluble olfactory proteins in insects. Biol. Rev. Camb. Philos. Soc..

[15] Hern A., Dorn S. (2002). Induction of volatile emissions from ripening apple fruits infested with *Cydia pomonella* and the attraction of adult females. Entomol. Exp. Appl..

[16] Vogt R.G., Riddiford L.M. (1981). Pheromone binding and inactivation by moth antennae. Nature.

[17] Fan J., Francis F., Liu Y., Chen J., Cheng D. (2011). An overview of odorant-binding protein functions in insect peripheral olfactory reception. Genet. Mol. Res..

[18] Zeng F.F., Zhao Z.F., Yan M.J., Zhou W., Zhang Z., Zhang A., Lu Z.X., Wang M.Q. (2015). Identification and comparative expression profiles of chemoreception genes revealed from major chemoreception organs of the rice leaf folder, *Cnaphalocrocis medinalis* (Lepidoptera: Pyralidae). PLoS ONE.

[19] El-Sayed A.M., Byers J.A., Manning L.M., Jürgens A., Mitchell V.J., Suckling D.M. (2008). Floral scent of Canada thistle and its potential as a generic insect attractant. J. Econ. Entomol..

[20] Szanyi S., Nagy A., Szarukán I., Varga Z., Jósvai J.K., Tóth M. (2022). A chemical lure for trapping both sexes of *Amata phegea* L.. Insects.

[21] Meagher R.L. (2003). Trapping noctuid moths with synthetic floral volatile lures. Entomol. Exp. Appl..

[22] Rainho H.L., Silva W.D., Gonçalves F.G., Savaris M., Bento J.M.S. (2022). Hexanal combined with decanal mediate host location by the bamboo powderpost beetle, *Dinoderus minutus*. Entomol. Exp. Appl..

[23] Zheng X.S., Lu Z.X., Xu H.X., Lu Y.H., Yang T.J., Tian J.C. (2021). An Attractant for Luring Both Female and Male Moths of Cnaphalocrocis medinalis and Its Application.

[24] Zhu A.X., Qiu Q., Liu X.D. (2015). A method for rearing the rice leaf folder (*Cnaphalocrocis medinalis*) using wheat seedlings. Chin. J. Appl. Entomol..

[25] Wei B., Gao H.Y., Zheng X.S. (2022). EAG responses of adult *Cnaphalocrocis medinalis* to plant volatiles. Chin. J. Appl. Entomol..

[26] Liu S., Wang W.L., Zhang Y.X., Zhang B.X., Rao X.J., Liu X.M., Wang D.M., Li S.G. (2017). Transcriptome sequencing reveals abundant olfactory genes in the antennae of the rice leaffolder, *Cnaphalocrocis medinalis* (Lepidoptera: Pyralidae). Entomol. Sci..

[27] Livak K.J., Schmittgen T.D. (2001). Analysis of relative gene expression data using real-time quantitative PCR and the 2^−ΔΔCT^ method. Methods.

[28] Bradford M.M. (1976). A rapid and sensitive method for the quantitation of microgram quantities of protein utilizing the principle of protein-dye binding. Anal. Biochem..

[29] Zeng F.F., Liu H., Zhang A., Lu Z., Leal W.S., Abdelnabby H., Wang M. (2018). Three chemosensory proteins from the rice leaf folder *Cnaphalocrocis medinalis* involved in host volatile and sex pheromone reception. Insect Mol. Biol..

[30] Scatchard G. (1949). The attraction of proteins for small molecules and ions. Ann. N. Y. Acad. Sci..

[31] Scala A., Allmann S., Mirabella R., Haring M.A., Schuurink R.C. (2013). Green leaf volatiles: A plant’s multifunctional weapon against herbivores and pathogens. Int. J. Mol. Sci..

[32] Fang Y., Zeng R., Lu S.F., Dai L., Wan X. (2018). The synergistic attractiveness effect of plant volatiles to sex pheromones in a moth. J. Asia-Pac. Entomol..

[33] Ochieng S.A., Park K.C., Baker T.C. (2002). Host plant volatiles synergize responses of sex pheromone-specific olfactory receptor neurons in male *Helicoverpa zea*. J. Comp. Physiol. A.

[34] Cattaneo A.M. (2018). Current status on the functional characterization of chemosensory receptors of *Cydia pomonella* (Lepidoptera: Tortricidae). Front. Behav. Neurosci..

[35] Zhou X., Wang Z., Cui G., Du Z., Qian Y., Yang S., Liu M., Guo J. (2022). Binding properties of odorant-binding protein 4 of *Tirathaba rufivena* to *Areca catechu* volatiles. Plants.

[36] Liu H., Duan H., Wang Q., Xiao Y., Wang Q., Xiao Q., Sun L., Zhang Y. (2019). Key amino residues determining binding activities of the odorant binding protein AlucOBP22 to two host plant terpenoids of *Apolygus lucorum*. J. Agric. Food Chem..

[37] Shields V.D., Hildebrand J.G. (2000). Responses of a population of antennal olfactory receptor cells in the female moth *Manduca sexta* to plant-associated volatile organic compounds. J. Comp. Physiol. A.

[38] Hillier N.K., Kleineidam C., Vickers N.J. (2006). Physiology and glomerular projections of olfactory receptor neurons on the antenna of female *Heliothis virescens* (Lepidoptera: Noctuidae) responsive to behaviorally relevant odors. J. Comp. Physiol. A.

[39] Kim M.S., Repp A., Smith D.P. (1998). LUSH odorant-binding protein mediates chemosensory responses to alcohols in *Drosophila melanogaster*. Genetics.

[40] Renwick J.A. (2001). Variable diets and changing taste in plant-insect relationships. J. Chem. Ecol..

[41] Tumlinson J.H., Yonce C.E., Doolittle R.E., Heath R.R., Gentry C.R., Mitchell E.R. (1974). Sex pheromones and reproductive isolation of the lesser peachtree borer and the peachtree borer. Science.

[42] Zhang Z., Zhang M., Yan S., Wang G., Liu Y. (2016). A female-biased odorant receptor from *Apolygus lucorum* (Meyer-Dür) tuned to some plant odors. Int. J. Mol. Sci..

[43] Chang H., Liu Y., Ai D., Jiang X., Dong S., Wang G. (2017). A pheromone antagonist regulates optimal mating time in the moth *Helicoverpa armigera*. Curr. Biol..

[44] Du Y., Chen J. (2021). The odorant binding protein, SiOBP5, mediates alarm pheromone olfactory recognition in the red imported fire ant, *Solenopsis invicta*. Biomolecules.

[45] Jia C., Mohamed A., Cattaneo A.M., Huang X., Keyhani N.O., Gu M., Zang L., Zhang W. (2023). Odorant-binding proteins and chemosensory proteins in *Spodoptera frugiperda*: From genome-wide identification and developmental stage-related expression analysis to the perception of host plant odors, sex pheromones, and insecticides. Int. J. Mol. Sci..

[46] Yang Y.T., Su Q., Shi L.L., Chen G., Zeng Y., Shi C.H., Zhang Y.J. (2019). Electrophysiological and behavioral responses of *Bradysia odoriphaga* (Diptera: Sciaridae) to volatiles from its host plant, Chinese chives (*Allium tuberosum* Rottler ex Spreng). J. Econ. Entomol..

[47] Zhu J., Wang F., Zhang Y., Yang Y., Hua D. (2023). Odorant-binding protein 10 from *Bradysia odoriphaga* (Diptera: Sciaridae) binds volatile host plant compounds. J. Insect Sci..

[48] Ullah R.M.K., Quershi S.R., Adeel M.M., Abdelnabby H., Waris M.I., Duan S.G., Wang M.Q. (2020). An odorant binding protein (SaveOBP9) involved in chemoreception of the wheat aphid *Sitobion avenae*. Int. J. Mol. Sci..

[49] Khuhro S.A., Liao H., Dong X.T., Yu Q., Yan Q., Dong S.L. (2017). Two general odorant binding proteins display high bindings to both host plant volatiles and sex pheromones in a pyralid moth *Chilo suppressalis* (Lepidoptera: Pyralidae). J. Asia-Pac. Entomol..

[50] Leal W.S., Ishida Y., Pelletier J., Xu W., Rayo J., Xu X., Ames J.B. (2019). Olfactory proteins mediating chemical communication in the navel orangeworm moth, *Amyelois transitella*. PLoS ONE.

[51] Newcomb R.D., Sirey T.M., Rassam M., Greenwood D.R. (2002). Pheromone binding proteins of *Epiphyas postvittana* (Lepidoptera: Tortricidae) are encoded at a single locus. Insect Biochem. Mol. Biol..

[52] Mao G.F., Tian J., Li T., Fouad H., Ga’al H., Mo J.C. (2018). Behavioral responses of *Anagrus nilaparvatae* to common terpenoids, aromatic compounds, and fatty acid derivatives from rice plants. Entomol. Exp. Appl..

[53] Hinge V., Patil H., Nadaf A. (2016). Comparative Characterization of Aroma Volatiles and Related Gene Expression Analysis at Vegetative and Mature Stages in Basmati and Non-Basmati Rice (*Oryza sativa* L.) Cultivars. Appl. Biochem. Biotechnol..

[54] Du H.T., Li Y., Zhu J., Liu F. (2022). Host-plant volatiles enhance the attraction of *Cnaphalocrocis medinalis* (Lepidoptera: Crambidae) to sex pheromone. Chemoecology.

[55] Paramita B., Chiranjit M., Adinpunya M. (2017). Enzymatic production and emission of floral scent volatiles in *Jasminum sambac*. Plant Sci..

