# Supplementary material for: Molecular Characterization of Plant Volatile Compound Interactions with Cnaphalocrocis medinalis Odorant-Binding Proteins
Source: Plants (Basel). 2024 Feb 7;13(4):479. doi: 10.3390/plants13040479 (PMC10892019; doi:10.3390/plants13040479)
Supplement: Supplementary file 1 [file plants-13-00479-s001.zip › plants-2815436-supplementary.pdf]

Table S1. RT-qPCR primer sequences of *Cnaphalocrocis medinalis* odorant binding proteins genes.

Primer	Sequence（5’-3’）	Size (bp)
β-Actin-F	CGAGCGTGGTTACTCATTCA	20
β-Actin-R	ATGACTTCTCGAGCGAGCTG	20
CmedGOBP1-F	TGATCCACAACTGCGAGAAG	20
CmedGOBP1-R	TCGGTCTGCATGATGAACTC	20
CmedGOBP2-F	GGTACTCTTGGGGCTGATGA	20
CmedGOBP2-R	TCCTCGTTCCAGAAATGCTT	20
CmedPBP1-F	TCTCCCAGACGCCATAAACA	20
CmedPBP1-R	TACTCGTGCGCATTTCCATG	20
CmedPBP2-F	TGGCCAATGTCAGAAGGAGT	20
CmedPBP2-R	CTGTCGTCTTGCCCTTATGC	20
CmedOBP1-F	GGGATGACAAGACAGCAGTTAA	22
CmedOBP1-R	TCATGTCCACCTGCTTCACC	20
CmedOBP6-F	ATTCGGCTGTTGGAGGAAGA	20
CmedOBP6-R	CGCTCGTGACTGTACATGTT	20
CmedOBP15-F	TTGGATCCTTGTTTCCTTGC	20
CmedOBP15-R	GGATCTCTCGCAGCCTTCTA	20
CmedOBP26-F	CGTAAGCATCGACGAGATCA	20
CmedOBP26-R	GCCCTCCTTACCATCAGACA	20

Table S2. RT-PCR primers for *Cnaphalocrocis medinalis* odorant binding protein genes.

Primer	Sequence (5’-3’)	Size (bp)
CmedGOBP1-F	AAAA*GGATCC*ATGCGCTTGGAGGCTGC	27
CmedGOBP1-R	TTT*AAGCTT*CTAGGCCTCGGTCTGCAT	27
CmedGOBP2-F	AAAA*GAATTC*GGGAGTGAAGGAACAGA	27
CmedGOBP2-R	TTT*AAGCTT*CATCATAATCAGCATCAA	27
CmedPBP2-F	AA*GGATCC*ATGTGGGCTAAAACG	23

CmedPBP2-R	TT*AAGCTT*CTACTGCATCTCAGCTACCA	28
CmedOBP1-L	AA*GGATCC*ATGGATGGAAAAAATCTTCC	28
CmedOBP1-R	TT*AAGCTT*TCAAGGAAATACGAAGTTTT	28
CmedOBP6-L	AA*GGATCC*ATGGGTTCCCCCTCATTCTC	28
CmedOBP6-R	TT*AAGCTT*TTAAGTTTTTGGCCGCCTG	28
CmedOBP15-L	AA*GAATTC*ATGTTGAAGTTGGTTTTCAC	28
CmedOBP15-R	TT*AAGCTT*TCACCACATTCCTGTACCAG	28
CmedOBP26-L	AA*GGATCC*ATGATGAAGTTTAATTTACT	28
CmedOBP26-R	TT*AAGCTT*TCAACGACGACGGGCTTCAG	28

The italic sequences are the restriction sites of *Bam*HI (GGATCC), *Eco*RI (GAATTC), and *Hind*III (AAGCTT).

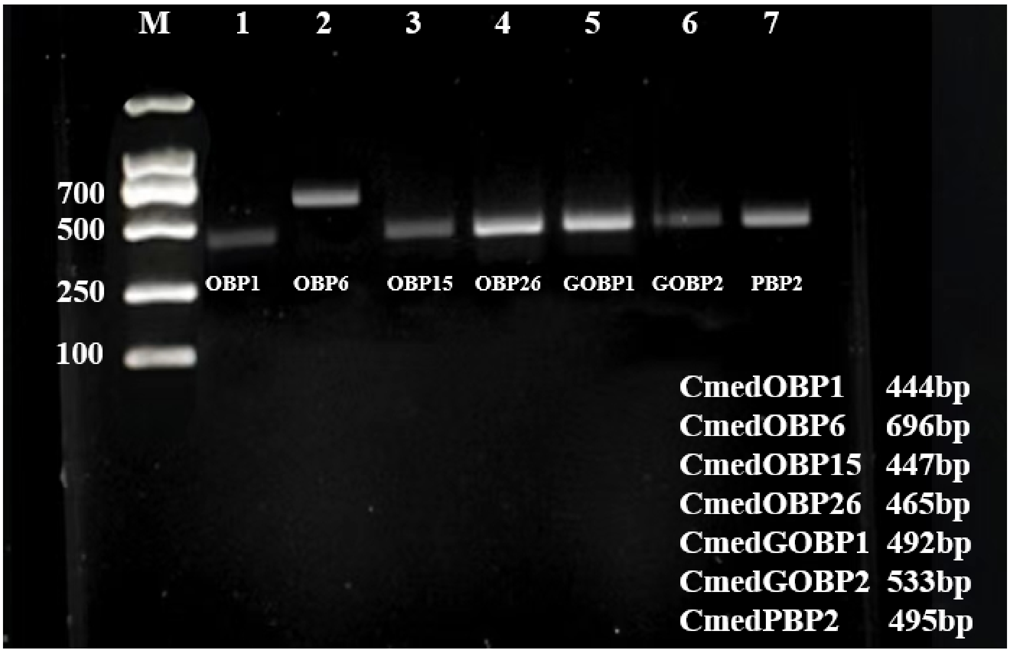

Figure S1. Identification of amplification bands of *Cnaphalocrocis medinalis* odorant binding proteins genes by agarose gel electrophoresis.

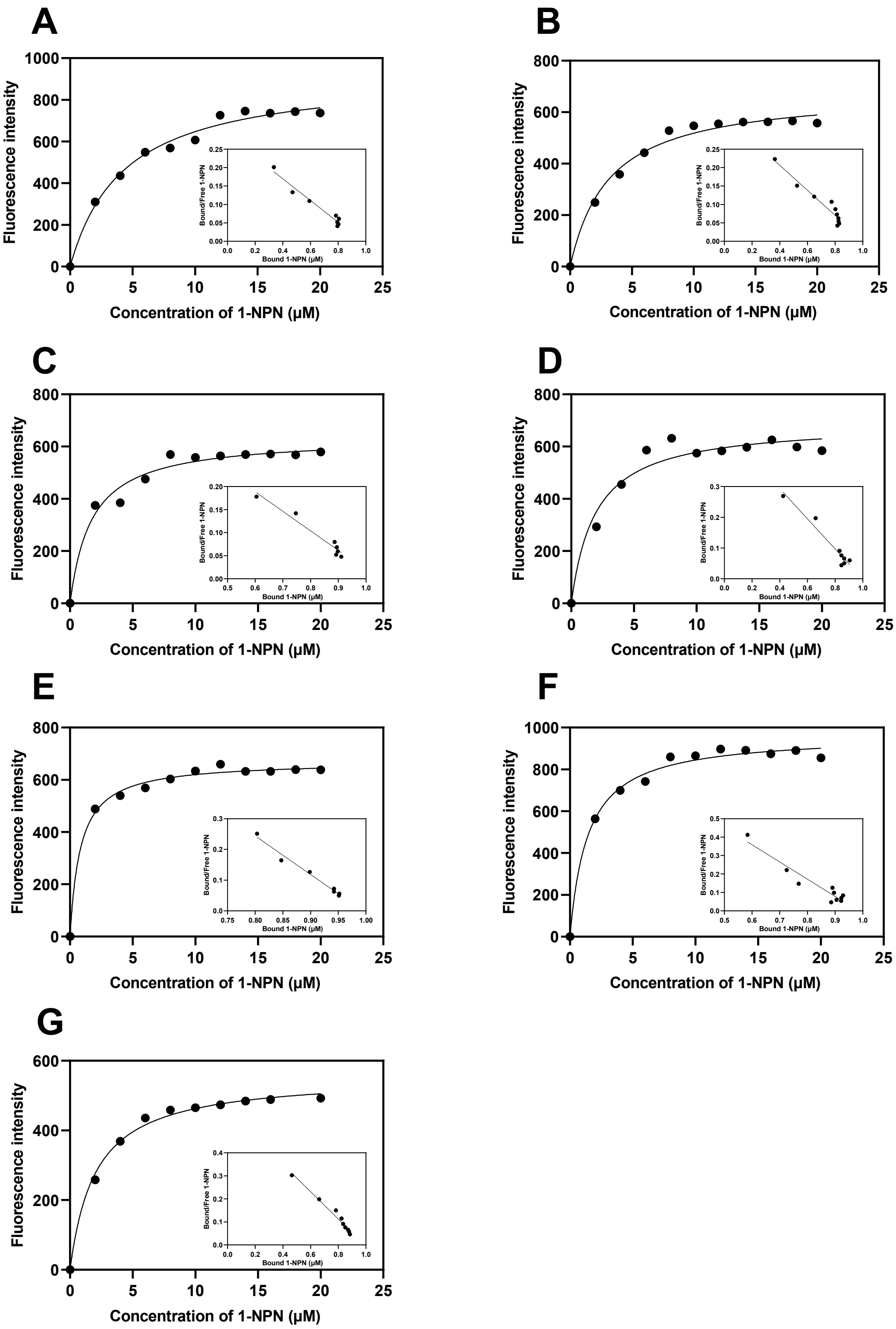

Figure S2. The binding curves and the Scatchard equations of N-phenyl-1-naphthylamine (1-NPN) and *Cnaphalocrocis medinalis* odorant binding proteins (CmedOBPs). A–G The binding curves and the Scatchard equations of 1-NPN and (**A**) CmedGOBP1, (**B**) CmedGOBP2, (**C**) CmedPBP2, (**D**) CmedOBP1, (**E**) CmedOBP6, (**F**) CmedOBP15, and (**G**) CmedOBP26.
